# Ruptured Mycotic Aneurysm of the Superior Mesenteric Artery: A Case Report

**DOI:** 10.7759/cureus.54004

**Published:** 2024-02-11

**Authors:** Sai Swarupa Vulasala, Anastasia Singareddy, Sarvika M Dasari, Max Wallack, Dheeraj Gopireddy

**Affiliations:** 1 Radiology, University of Florida College of Medicine – Jacksonville, Jacksonville, USA; 2 Skin Biology and Dermatological Sciences, University of Miami Miller School of Medicine, Miami, USA; 3 Health Sciences, Florida State University, Tallahassee, USA

**Keywords:** hiv-positive, contrast-enhanced ct of the abdomen and pelvis, body mri, infected aneurysm, mesenteric vasculitis, superior mesenteric artery aneurysm

## Abstract

An infected (mycotic) aneurysm of the visceral arteries is an uncommon entity, which may arise from a secondary infection of a preexisting aneurysm or be due to degeneration from a primary infection. Mycotic aneurysms require prompt recognition and definitive treatment; otherwise, there can be devastating morbidity and mortality. We present the case of a 51-year-old female with HIV and Crohn's disease who presented with subacute abdominal pain, nausea, and vomiting and was found to have an ultimately fatal mycotic aneurysm of the superior mesenteric artery. In addition, we discuss the characteristic imaging features of mycotic aneurysms on computed tomography and magnetic resonance imaging.

## Introduction

Mycotic aneurysm refers to the dilation of an infected arterial wall and was first described by William Osler in 1885 [1]. Following the femoral artery (38%) and the abdominal aorta (31%), visceral arteries are the third most commonly involved vessel, accounting for 9% of reported cases [2]. Among the visceral arteries, mycotic aneurysms are more frequently seen in the superior mesenteric artery (SMA) [3,4]. Here, we discuss a patient who suffered a fatal rupture of a mycotic SMA aneurysm. In this case, we aim to guide clinicians in the prompt management of mycotic SMA aneurysms, which remains associated with high morbidity and mortality. 

## Case presentation

A 51-year-old female presented to the emergency department with a four-day history of nausea, vomiting, and abdominal pain. Her medical history includes HIV infection with unknown viral load, CD4 count, or use of antiretroviral therapy, Crohn's disease status post ileostomy which had since been reversed, hypertension, intravenous drug use, and active tobacco use. On presentation, she was alert and endorsed persistent abdominal pain and nausea. Upon further discussion, she stated that she had been experiencing several days of night sweats, cough, and subjective fevers. Her review of symptoms was otherwise negative. The patient was hypertensive on physical examination, with otherwise normal vital signs. Her physical exam revealed multiple healed scars from previous abdominal surgeries and was otherwise unremarkable. Laboratory analysis revealed mild anemia, without leukocytosis or metabolic derangement. Hepatic function and lipase were within normal limits. Further investigation revealed elevated erythrocyte sedimentation rate, C-reactive protein, complement 3, and complement 4. The patient was positive for HIV, hepatitis B surface antibody, and hepatitis B core antibody. Blood cultures grew no colonies, and she tested negative for hepatitis viral load, QuantiFERON, and syphilis. The urine drug screen was positive for cocaine. Rheumatologic testing, including antinuclear antibody (ANA), anti-neutrophil cytoplasmic antibodies (ANCA) panel, and rheumatoid factor (RF), were negative. The urine pregnancy test was negative.

**Figure 1:**
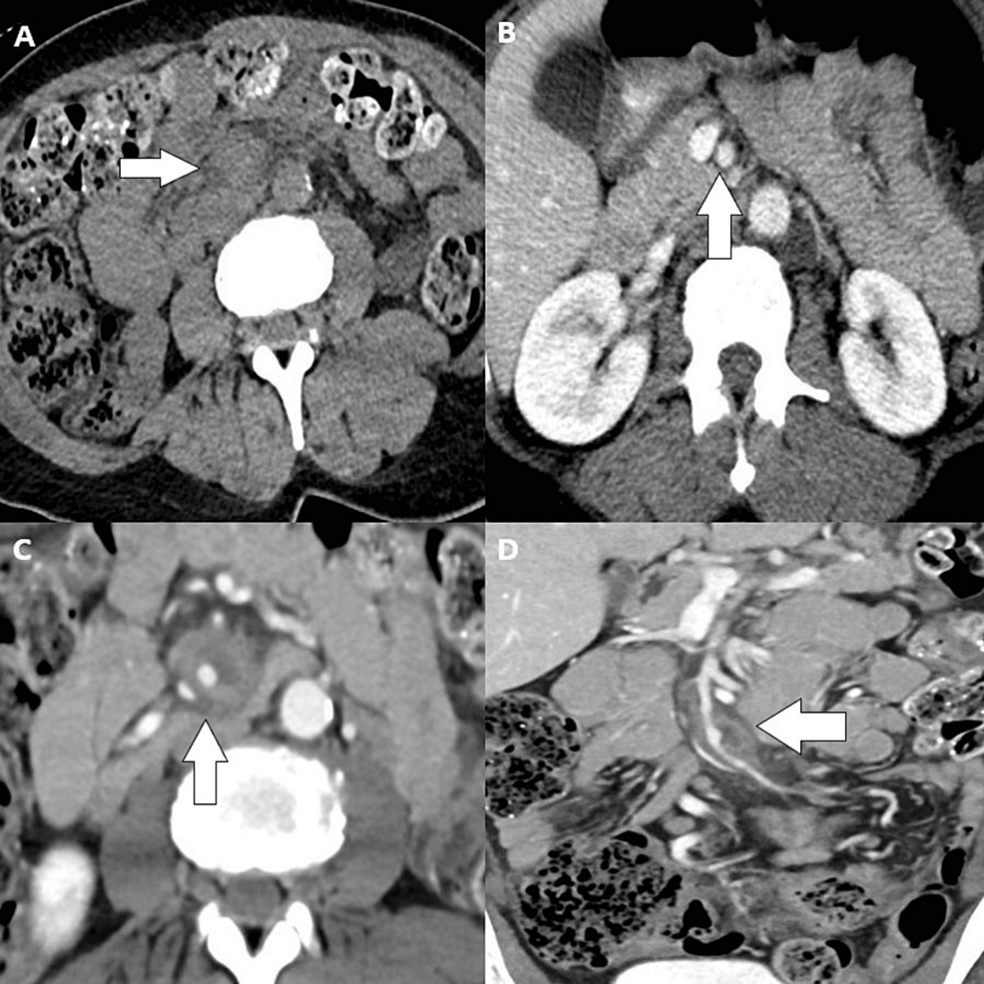
Composite figure of CT findings A. Noncontrast CT of the abdomen and pelvis. The arrow denotes nonspecific stranding around the SMA. B. Intravenous contrast-enhanced CT demonstrates normal proximal SMA at the arrow. C. Distal to normal-caliber SMA. The arrow denotes circumferential wall thickening and adjacent fat stranding. There was no evidence of complex fluid collection surrounding the disease segment of the vessel. D. Coronal reformat shows the extent of involved dilated and infected SMA (arrow). CT: computed tomography; SMA: superior mesenteric artery

Initial noncontrast computed tomography (CT) of the abdomen and pelvis showed nonspecific stranding around a short mid-segment of SMA. Contrast-enhanced CT further revealed circumferential thickening of the mid-segment of SMA, with adjacent mesenteric stranding raising concern for focal vasculitis (Figure 1). 

**Figure 2:**
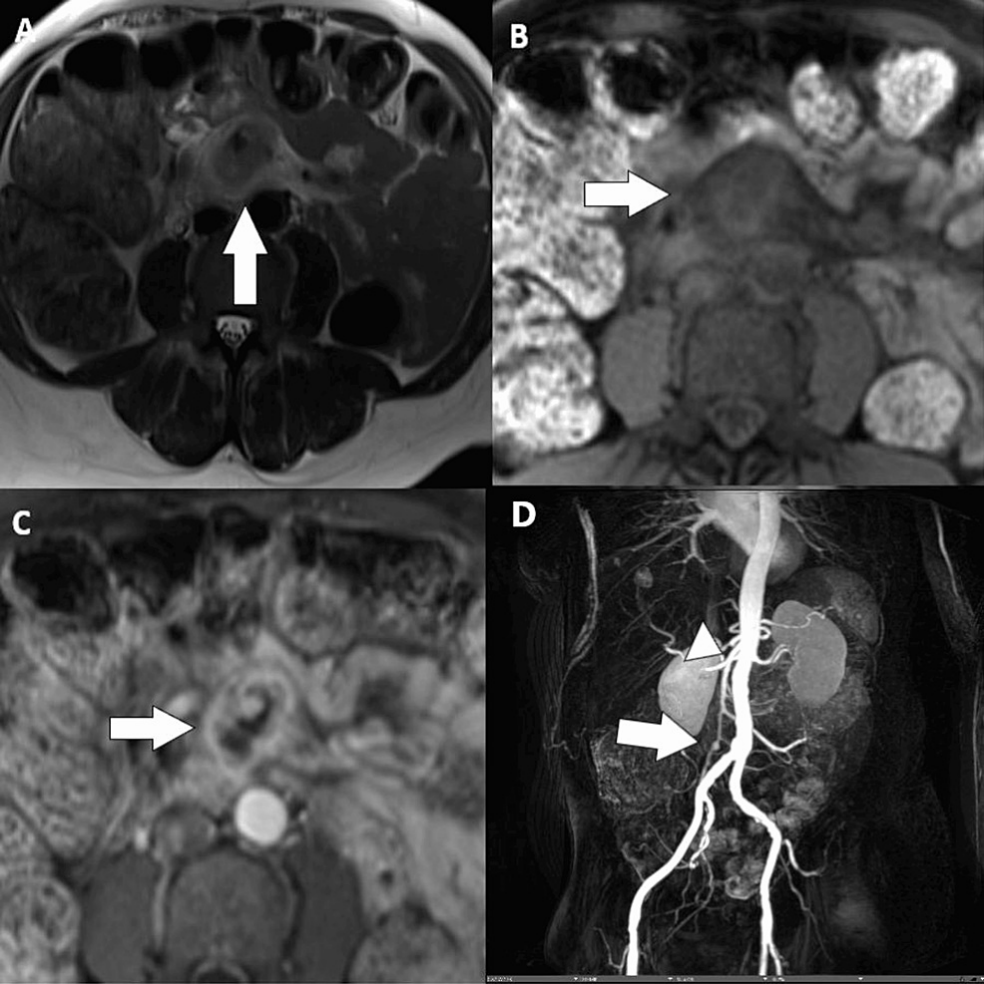
Composite findings of MRI A. MRI of the abdomen and pelvis. Single-shot fast spin-echo sequence. The arrow demonstrates wall thickening and stranding of the SMA. B. Precontrast VIBE sequence at the level of the mycotic aneurysm. The arrow reveals mural edema. C. Postcontrast VIBE sequence. The arrow denotes circumferential enhancement of the SMA wall. D. Coronal postcontrast VIBE MIP demonstrates normal-caliber proximal SMA (arrowhead) with foci of luminal dilation (arrow) and narrowing of the involved SMA, without additional aneurysm of the arterial system within the abdomen or pelvis. MRI: magnetic resonance imaging; SMA: superior mesenteric artery; VIBE: volumetric interpolated breath-hold examination; MIP: maximum intensity projection

Following the initiation of empiric antibiotic therapy, a clinical discussion was made for further treatment options, and magnetic resonance imaging (MRI) of the abdomen with and without contrast was requested for elucidation before definitive treatment. MRI revealed an irregularly thickened and edematous arterial wall (Figure 2), along with marked enhancement of the arterial adventitia which corroborated the superimposed infection of the SMA aneurysm. Due to the location of the mycotic aneurysm, nonoperative interventions were felt to carry high risk without the benefit of definitive treatment, and the patient deferred possible operative management.

The patient remained stable until the sixth day of hospitalization when the patient's clinical status suddenly deteriorated with hypotension and rapid distension of the abdomen. Prompt computed tomography angiography (CTA) demonstrated rupture of distal SMA aneurysm. The patient experienced cardiac arrest with the return of spontaneous circulation, and following a massive blood transfusion, she was brought for emergent laparotomy. Upon entering the abdomen, there was a massive hemoperitoneum secondary to ruptured distal SMA. During the operation, she continued to receive numerous blood products (in total, 29 blood products along with 4 liters of crystalloids in the operating room). Following the placement of a partial resuscitative endovascular balloon occlusion of the aorta (REBOA), vascular surgery achieved proximal SMA control, and inspection of the aneurysmal cavity demonstrated a completely destroyed arterial wall. Unfortunately, no branches within the aneurysmal sac were amenable to reconstruction due to diminutive size and friability due to inflammation. Direct inspection of additional vessels during vascular exploration revealed the involvement of the ileocolic branch, leading to the long-segment infarcted and non-viable small bowel. The patient remained unstable with continuous coagulopathic bleeding, complicated by acidosis and hypothermia. Unfortunately, further operative management was determined to be surgically futile, and further resuscitation attempts were aborted. Her next of kin declined the offer for an autopsy to reveal the underlying cause of her demise.

## Discussion

Aneurysmal involvement of the visceral arteries is a rare condition that constitutes 0.1-2% of all aneurysms [5,6]. The splenic and hepatic arteries are more commonly involved and represent 60-80% and 20% of visceral aneurysms, respectively [7-9]. In contrast, SMA aneurysms represent only 5.5-8.6% of visceral aneurysms [10,11]. Although uncommon, SMA aneurysms are associated with a high rate of rupture (38-50%) and mortality (25-100%) depending on their size and location [10,11]. Our patient presented with a distal SMA aneurysm complicated by infection with subsequent fatal rupture. The etiology is presumed to be infectious in the setting of risk factors, including intravenous drug use and chronic immunosuppression due to HIV/AIDS [4]. Infection is the least common cause of SMA aneurysms constituting only 0.7-3% of all aneurysms, and 18-50% of patients demonstrate negative cultures, similar to our patient [7,10]. If positive, cultures usually grow streptococcal or staphylococcal species [6]. Mycotic aneurysms result from the invasion of microbial organisms into the vessel wall, resulting in their destruction and dilation [7]. Other causes of SMA aneurysms include hypertension, atherosclerosis, rheumatologic conditions, Ehlers-Danlos syndrome, and vascular damage secondary to diabetes mellitus [12]. 

Patients typically present with a classical pentad of abdominal pain, fever, malaise, weight loss, and anorexia. Without prompt intervention, the patient's clinical status may rapidly progress to sepsis or rupture [7]. CTA has replaced catheter angiography as the standard modality of choice to detect aneurysms and delineate the anatomy for more precise surgical intervention [13] due to decreased morbidity as well as improved spatial resolution. Magnetic resonance angiography (MRA) is an acceptable alternative modality; however, it is limited by its longer scan duration and susceptibility to motion artifacts. Early diagnostic clues for mycotic aneurysms include irregular arterial walls, soft-tissue mass or fat stranding, and peri-or intravascular gas [4]. Fat stranding and adjacent soft-tissue density mass are common findings observed in approximately 48% of patients [14]. If there is necrosis of the vessel wall, there can be heterogeneity of the perivascular soft tissues [4]. The rupture of the aneurysm can be confirmed with contrast extravasation. On MRI, perivascular edema and soft-tissue mass are hypointense on T1-weighted and hyperintense on T2-weighted sequences (Figure 2) [4]. There can be diffusion restriction and enhancement of the adventitia due to increased cellularity. 

The management of mycotic SMA aneurysms includes medical, endovascular, and surgical therapies. Medical management alone is insufficient to prevent progression to aneurysmal rupture, which has a high mortality rate of 50% [6,7]. Hence, excision of the aneurysm has become standard practice, while endovascular options are reserved for poor surgical candidates [7]. Aneurysmectomy, or surgical aneurysmal resection, has been studied, sometimes including vessel reconstruction, and employed in previous case studies. Performing aneurysmectomy alone may result in the interruption of distal vascular flow and is reported to be associated with bowel ischemia and bowel resection in 10% of cases [7]. Kordzadeh et al. demonstrated that the rate of bowel resection is high with aneurysmectomy alone, whereas patients had excellent outcomes when surgical revascularization was performed with the great saphenous vein [13]. Therefore, vessel reconstruction must be considered if the affected SMA trunk or branches supply a substantial portion of the bowel. All ruptured mycotic SMA aneurysms should be repaired urgently using a combined surgical and endovascular approach. The latter is helpful for prompt proximal vessel control as was attempted in the above case.

Endovascular therapies are also considered for patients with SMA aneurysms in anatomically feasible regions [15]. Flow-diverting stents have recently been used to divert blood flow into side branches, thereby allowing shrinkage or thrombosis of the aneurysmal sac, which has been reported in 90% of cases [16]. However, these stents are limited by cost and require the target artery diameter to be at most 5-5.5 mm [12]. In addition, the presence of a stent in hostile tissue worsens microbial colonization and infection [13]. Delaying the endovascular procedure until negative blood cultures, using antibiotic-coated stents, and performing adjunctive debridement have better results in mycotic aneurysms; however, more extensive studies are required to validate these techniques [17].

## Conclusions

Patients with suspected mycotic SMA aneurysms should promptly undergo surgical intervention following stabilization to prevent rupture and reduce mortality risk. If treatment is deferred or not pursued, outcomes can be catastrophic. Previously, aneurysmectomy with or without reconstruction has proven effective, although further studies are mandatory to validate the use of routine endovascular repair.
